# Arsenic-induced instrumental genes of apoptotic signal amplification in death-survival interplay

**DOI:** 10.1038/cddiscovery.2016.78

**Published:** 2016-10-17

**Authors:** Sonali Roy, Bardwi Narzary, Atish Ray, Manobjyoti Bordoloi

**Affiliations:** 1 Natural Product Chemistry Group, CSIR-NEIST, Jorhat, Assam 785006, India; 2 Immunobiology Laboratory, Department of Zoology, University of Delhi, Delhi 110007, India

## Abstract

Arsenic is a global health concern at present and it is well reported for causing systemic toxicity. It is also well known for generation of free radical and inducing apoptosis in different cell types. Paradoxically arsenic is reported to be a susceptible carcinogen as well. There are several reports demonstrating diverse mechanism of apoptosis in different cell types. However, the universal scenario of instrumental genes and their interaction leading to amplification of apoptotic signal are yet to be completely uncovered, which is predicted here. Conventional studies on signaling pathway aided by time and concentration kinetics data are inadequate for prediction of anchored genes for apoptotic signal amplification. Therefore, expression profile-based approach is adopted. Core apoptosis related and glutathione metabolism genes in 1 and 10 *μ*M of arsenic-treated HepG2 cells were analyzed after 12 h of incubation. An arsenic treatment of 1 *μ*M exhibits no cell death at 12 h, whereas 10 *μ*M arsenic treatment reveals around 50% cell death at 12 h. Results depict 28 and 44 affected genes in 1 and 10 *μ*M arsenic-treated cells, respectively. Early initiation of apoptotic signaling is detected in no cell death regimens (at 1 *μ*M), whereas amplified apoptotic signal is demonstrated at 50% cell death regimens (at 10 *μ*M). Instrumental genes involved in progression of apoptosis in the concourse of cell death and survival is designated from the responsive genes common to both the condition. We predict the initiation process is fairly aided by the activation of intrinsic pathway, which is amplified via TNF signaling and extrinsic pathway. Furthermore, regulatory genes involved in interplay between apoptosis/anti-apoptosis and their interactions are demonstrated here.

## Introduction

Arsenic is a well-known environmental contaminant causing systemic toxicity.^1,2^ Paradoxically, it is also a susceptible carcinogen. This contrasting behavior of arsenic is fairly concentrated and cell-type dependent. Chronic- and low-concentration exposure has been reported to be responsible for carcinogenesis but not apoptosis,^3,4^ whereas high-concentration arsenic is responsible for apoptosis.^5^ Arsenic-associated detrimental effects also include generation of reactive oxygen species and lipid per oxidation.^6^ Manifestation of apoptosis triggered by arsenic is well studied in liver.^5,7–10^ In normal hepatocytes, apoptosis is evidently detrimental. Contrastingly in carcinoma cells, apoptosis is a potential tool for growth inhibition and therefore arsenic has been used as chemotherapeutic agents for treating certain types of cancer, including acute promyelocytic leukemia.^11^ In the last decades, it has been shown to share diverse apoptotic pathway depending on exposure concentration and particular cell type.^8–10^ It has also been established that arsenic-induced apoptosis is free radical mediated. Moreover, glutathione dynamics determine the major fate of the cell.^5^

In the present context, arsenic is selected as a well established inducer of apoptosis. A number of studies determined the different pathways of apoptosis in different cell types. However, there is hardly any concluding report predicting the key molecules influential for progression of apoptosis and amplification apoptotic signals. Targeting a single gene or pathway intermediate leading to apoptosis is inadequate for forecasting the instrumental molecules. Furthermore, time kinetics provide substantial information on magnitude of expression of certain genes or proteins in course of time and predicts predominate signaling event thereof which is technically not enough to anticipate interaction between genes or molecules involved in the initiation and amplification of apoptotic signals. We hypothesize that the complete apoptotic procedure is divided into three phases, including initiation, progression and commencement, thus the initiation process can be reflected in ‘no cell death’ regimen, whereas commencement of apoptosis can be demonstrated in ‘50% cell death’ sector. Taking together, the signal amplification mechanism can be predicted. Therefore, we adopted an expression profile-based approach and report here the possible regulatory genes and their interaction leading to progression of apoptosis in the concourse of death and survival in HepG2 cells.

## Results

### MTT cell viability assay

MTT cell viability assay demonstrates an overall time-dependent increase in percent cell death. Concentration-dependent cell death is most significant from 30 min onwards and most prominent in the low-concentration regimens. Early incubation time (15 min) does not demonstrate changes in percentage of cell death in any of the treatment concentration as compared with control. On the other hand, maximum cell death was observed in 10 *μ*M treatment concentration at 6 h onwards attaining the peak of around 50%. However, changes in cell death among 6, 12 and 24 h of incubation period are not significant (Supplementary Data 1).

### Detection of apoptosis

**Figure 1:**
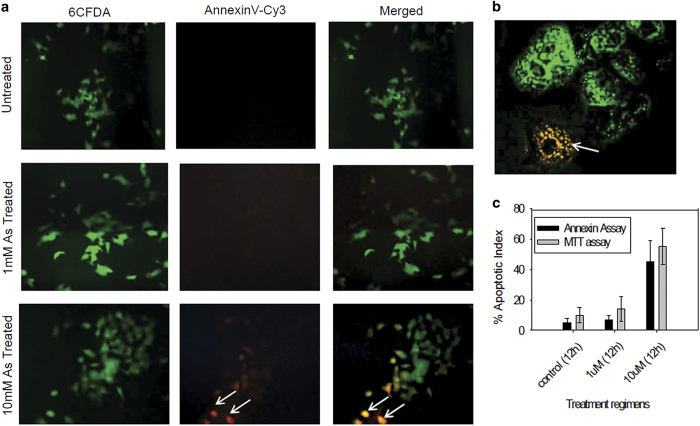
(**a**) Annexin V-Cy3/6CFDA dual staining of HepG2 cells exposed to 1 and 10 *μ*M arsenic trioxide with untreated control. Arsenic-treated cells of 1 *μ*M demonstrate no significant changes as compared with control, whereas 10 *μ*M arsenic-treated cells exhibit considerable number of apoptotic cells represented by Annexin V-Cy3/6CFDA dual-positive cells, which appears yellow-orange in the merged panel. (**b**) Typical apoptotic (dual-positive) and non-apoptotic (only 6CFDA positive) cell in higher magnification. (**c**) Apoptotic index calculated from comparisons of percent cell death revealed by MTT assay as well as percent apoptotic cells obtained from Annexin assay. Arrows represent the apoptotic cells.

Type of cell death was verified with Annexin V-Cy3/6CFDA dual staining of control and treated cells. 6CFDA (appears green) stained the viable cells, whereas, externalization of phosphatidyl serine was demonstrated by Annexin-conjugated Cy3 staining. Therefore, apoptotic cells appear yellowish orange in the merged image for being dual-positive (6CFDA appears green and Cy3 appears red). Live cells are only 6CFDA positive, which appears green. One micromolar treatment depicts existence of hardly apoptotic and/or necrotic cells. On the other hand, 10 *μ*M treatment concentration depicts more than 45% apoptotic cells demonstrated by Anexin V-Cy3/6CFDA dual-positive cells (Figure 1a). The result corroborates with our MTT-cytotoxicity data. Typical non-apoptotic and apoptotic cells are represented in the merged image of higher magnification (Figure 1b). Figure 1c expresses apoptotic index demonstrating a comparative account of percent cell death obtained from MTT result and percent apoptosis cells obtained from Annexin/CFDA assay.

### Expression profile of apoptotic genes

**Figure 2:**
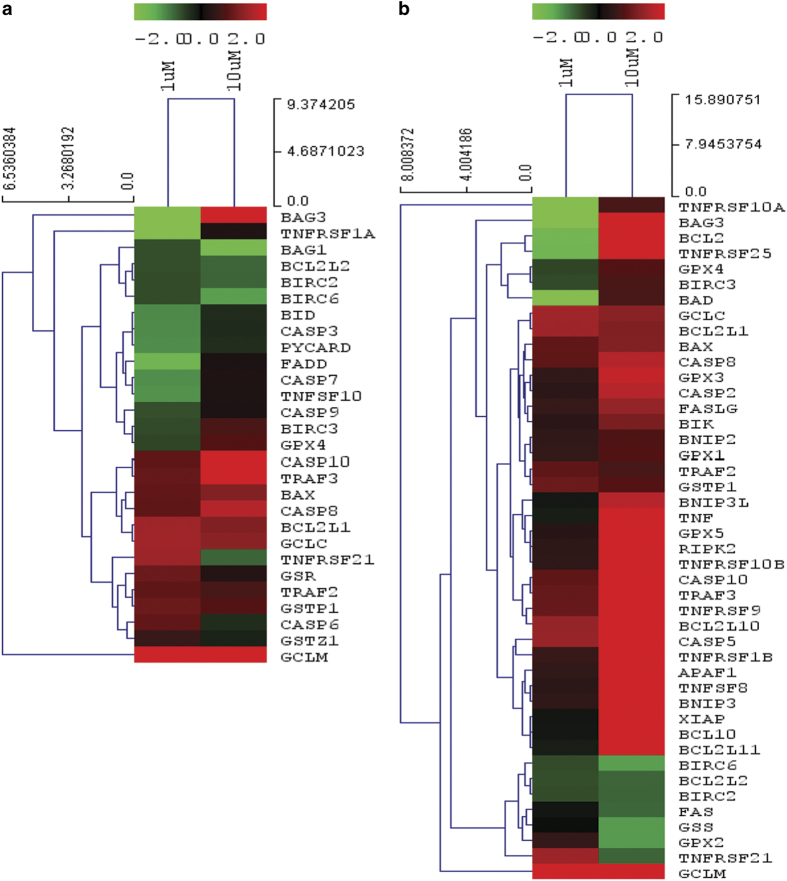
Heat maps and hierarchical clusters of core apoptosis-related and glutathione metabolism-related genes up- and downregulated significantly in 1 *μ*M (**a**) and 10 *μ*M (**b**) arsenic-treated cells as compared with control. An arsenic treatment of 1 *μ*M demonstrates 15 downregulated and 13 upregulated genes, whereas 10 *μ*M treatment of arsenic-treated cells exhibits 37 upregulated and 7 downregulated genes as compared with control.

**Figure 3:**
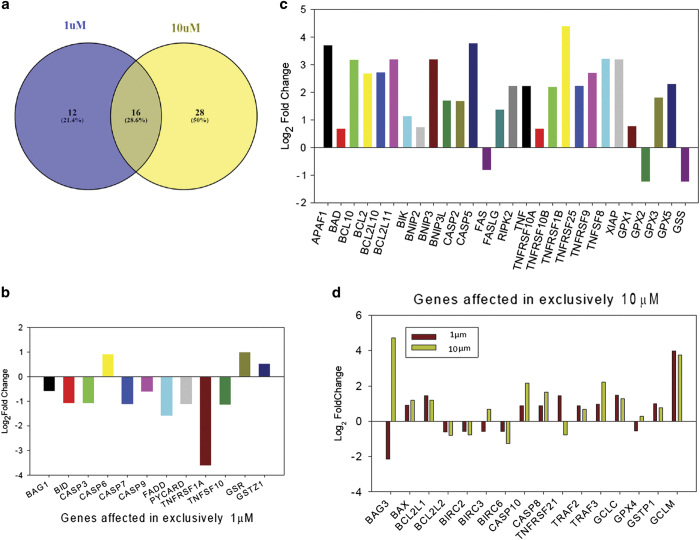
(**a**) Venn diagram representing the overlap between the genes affected in 1 and 10 *μ*M arsenic-treated cells. Twelve genes are affected (up/downregulated) exclusively at 1 *μ*M and 28 genes are affected exclusively in 10 *μ*M arsenic-treated cells. Sixteen genes are affected commonly in both the treatment concentrations. Magnitude of up/downregulated genes in 1 *μ*M (**b**), 10 *μ*M (**c**) and both (**d**) treatment concentration.

**Figure 4:**
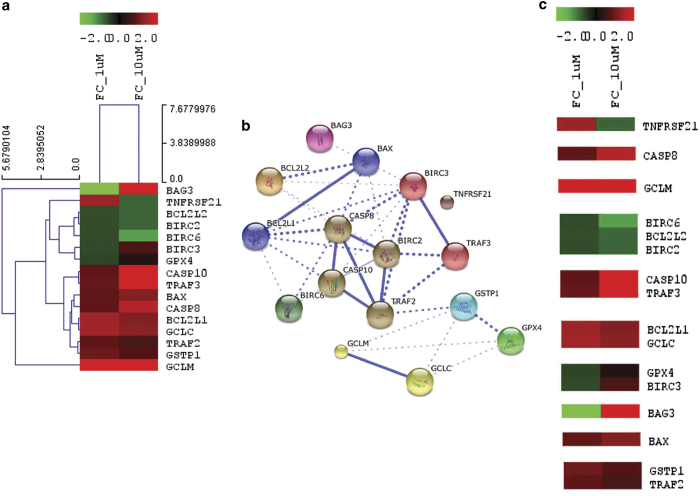
(**a**) Hierarchical clusters of commonly affected genes demonstrating the closeness of association among instrumental genes of apoptotic signal amplification (**b**) K means cluster of STRING database predicted gene interaction network and (**c**) K means cluster of experimentally derived of commonly affected genes in both treatment concentrations.

Our qPCR array results demonstrate significant up- or downregulation of core apoptosis-related genes, including caspases, caspase regulator, inhibitor of apoptosis, mitochondrial regulator of apoptosis and glutathione metabolism-related genes. Heat maps and hierarchical clusters demonstrate 28 affected genes in 1 *μ*M treatment concentration. Fifteen genes were found to be downregulated and 13 are upregulated among them (Figure 2a). On the other hand, at 10 *μ*M treatment concentration, 37 genes were found to be upregulated, whereas 7 were downregulated (Figure 2b). From the expression profile data, it is clear that 12 genes were found to be affected only at 1 *μ*M treatment concentration (3 upregulated and 9 downregulated). Similarly, 28 genes are affected at 10 *μ*M treatment concentration exclusively of which 3 are downregulated and 25 are upregulated (Figures 3a–c). Thereby, 16 genes are found to be affected commonly in both the treatment concentration. Figure 4 depicts the hierarchical cluster (Figure 4a) and K means cluster of commonly affected genes in both treatment concentrations demonstrating the close association thereof (Figures 4b and c).

## Discussion

We have selected HepG2 as cell type to demonstrate the said hypothesis. There is obviously difference in opinion regarding advantage of normal hepatocyte over cell line. In many of the aspects, normal hepatocytes were shown to be superior to the immortal cell lines.^12^ However, considering certain parameters, including easy availability, handling and synchronous response, HepG2 is considered as a tractable model for the present purpose. Moreover, HepG2 will provide an insight in respect of hepatocellular carcinoma as well.^13,14^

Our MTT cell viability assay demonstrates time- and concentration-dependent increase in cell death not exceeding 50% of the control (Supplementary Data 1). At 1 *μ*M treatment concentration, up to 12 h, there was no significant cell death; on the other hand, ~50% cell death was observed in case of 10 *μ*M arsenic treatment at 12 h onwards. Logarithmic concentration or dose shift is a successful measure to understand dose response data.^15^ Hence from our concentration-dependent cell death data, two treatment concentrations were selected in a span of log_10_ base shift (1 and 10 *μ*M) considering no cell death and 50% cell death regimens. Twelve hour treatment regimens were taken for further analysis. Annexin/CFDA staining of HepG2 cells confirm apoptosis (Figure 1).

From our qPCR-gene expression array data, we have clustered significantly affected core apoptosis-related genes. Hierarchical clusters demonstrate closely related genes at 1 and 10 *μ*M treatment concentration (Figures 2a and b). The diversification of pathway during progression of apoptosis is evident from our result. According to our hypothesis, pathway involved in initiation event of apoptosis was demonstrated by the affected genes in no cell death regimen, therefore, exclusively up- or downregulated genes in 1 *μ*M arsenic-treated cells were categorized as initial apoptotic signaling genes. Similarly, affected genes at 10 *μ*M treatment concentration depict the amplified apoptotic signals where about 50% cell death is apparent. Hence we predict that the genes common to both the concentration are instrumental genes (or molecules) responsible for amplification of apoptototic signals (Figure 3). Our results demonstrate *bag1, bid, casp3, casp6, casp7, casp9, fadd, pycard, tnfrsf1a, tnfsf10, gsr* and *gstz1* are affected in 1 *μ*M treatment concentration. All the genes except *casp6, gsr *and *gstz1* were downregulated. Among these genes, it is supposed that *bag1* downregulation, which is reported to act cooperatively with anti-apoptotic bcl2 (Entrez Gene ID 573, 2010) and caspase 6, a direct activator of caspase 8^16^ initiates the apoptotic signals. On the other hand, *gsr* and *gstz1* promote cell survival via glutathione balance. In support of our finding, earlier study demonstrates that GSR enzyme activity is responsible for apoptosis in rat hepatocytes,^5^ whereas GST is responsible for glutathione-mediated detoxication of arsenic.^17^ However, no single report can be cited in support of isoform-specific detoxication of arsenic because isoform-specific GST response depends on cell type and exposed chemicals.

Further we demonstrate that here amplified apoptotic signal is operated majorly via extrinsic pathway involving significant upregulation of TNF signaling cascades (*traf2 *and* traf3*) converging in *casp8* and *casp10* activation.^18,19^ Although, *tnfrsf21* was found to be downregulated in higher treatment concentration, cooperative role of other isoforms in promotion of apoptosis is evident. Death-survival interplay is apparent through anti-apoptotic and apoptotic mitochondrial regulators and components of glutathione pathways (*bag3, bax, bcl2l1, bcl2l2, birc2, birc3, birc6, gclc, gpx4, gstp1 *and *gclm*). Among these, increase in *bax* level certainly plays a critical role in maturation of apoptotic signal at the end. Because of persistent significant crosstalk between death and survival, it is too arbitrary to designate the molecules responsible for promotion of apoptosis in a stand-alone way. Hence the cooperatively acted genes in the tug of war between cell death and survival are hypothesized. Additionally, involvement of *bad*, *gpx2 *and *gss* is concomitant with *casp2* and *casp5* expression at the expanded phase of apoptosis. *Bad* is the pro-apoptotic member of mitochondrial regulation of apoptosis, whereas *gpx2* and *gss* are known to be involved in glutathione-dependent detoxification and glutathione biosynthesis, respectively. Figure 4 depicts the predicted anchored genes and there interaction essential for apoptosis progression.

## Conclusion

The competitive and cooperative response of pro-apoptotic and anti-apoptotic axis always exists during chemical exposure and any single molecule is not responsible to determine the fate. The present report designates the anchored cluster of genes predictably involved to direct the pathway toward apoptosis in the concourse of cell death and survival. It is concluded that intrinsic pathway is the initial process of apoptosis found to be responsive exclusively at low-treatment concentration (1 *μ*M), which in general remains subdued at no cell death condition. During apoptotic boom, extrinsic pathways are activated. There are several reports on crosstalk of extrinsic and intrinsic pathways but hardly any concluding information regarding the interaction of anchored molecules involved in progressive apoptotic pathways. We designate the genes, which are instrumental in progression of apoptosis. We anticipate that our report will provide a novel outlook in targeting molecules for inducing apoptosis in hepatocellular carcinoma as well as inhibiting apoptosis in normal cell.

## Materials and Methods

### Culture and maintenance of cell lines

HepG2 cells were procured from Sigma-Aldrich and cultured in complete media, minimum essential medium eagle supplemented with 10% fetus bovine serum, 10% penstrep (Sigma-Aldrich Co. LLC, St Louis, MO, USA) and 1% gentamycin (Sigma-Aldrich Co. LLC), and incubated under in 37°C humidified 5% CO_2_ atmosphere. After confluence is achieved, cells (1×10^6^ per ml) were seeded in tissue culture grade 96 well plates (Nunc, Thermo Fisher Scientific Inc., Waltham, MA, USA) for MTT assay and 6 well plates for RNA preparation in complete medium and incubated. After 24 h, the complete medium was replaced with fetus bovine serum free medium and incubated overnight. The cells were then treated with As_2_O_3_ in different concentrations (0.5, 1, 2, 5 and 10 *μ*M) into each well and incubated for different time period (15 min, 30 min, 1 h, 2 h, 4 h, 6 h, 12 h and 24 h). Cell maintained in arsenic-free medium served as control.

### Cytotoxicity (MTT) assay

The cytotoxicity was evaluated by *in vitro* MTT assay. The assay was performed according to the manufacturer’s guideline (Merck Millipore Corporation, Darmstadt, Germany). After termination of treatments, 10 *μ*l of MTT (5 mg/ml) was added to each well containing control and treated cells, mixed gently and incubated for 4 h. After incubation period, cells were observed under an inverted microscope for the presence of dark purple formazan crystals at the bottom of the wells. An aliquot of 0.1 ml isopropanol with 0.04 N HCl was added to each well and mixed thoroughly by repeated pipetting. Isopropanol dissolves the formazan to give a homogeneous blue solution suitable for absorbance measurement. The HCl converts the phenol red of tissue culture medium to a yellow color that does not interfere with MTT formazan measurement. The absorbance was measured on an ELISA plate reader (FilterMax F3 Multi-Mode Microplate Reader, Molecular Devices, Sunnyvale, CA, USA) with a test wavelength of 570 nm and a reference wavelength of 630 nm. All experiments were performed in triplicate. Cell death was expressed in percent of control.^20^

### Assessment of apoptosis by Annexin V-Cy3 staining

Annexin V-Cy3 staining was performed using Annexin V-Cy3 Apoptosis Detection Kit (Sigma) following the manufacturer’s guideline. Briefly, after the incubation period, cells were labeled with double-staining solution (AnnCy3 and 6CFDA) for 10 min and observed under microscope (Motic AE31). Image of the same field was captured with appropriate filters.

### qPCR array

RT profiler qPCR array plates for apoptotic and oxidative stress were procured from SA Bioscience, QIAGEN (Frederick, MD, USA) and gene expression profile was studied with Applied Biosystem 7500 real-time PCR system. Data were analyzed using relative fold change (2^−ΔΔCT^) as compared with control using six housekeeping genes and no template control. Significant up- or downregulated genes were selected considering log_2_ fold change value ⩾0.5 or ⩽−0.5 and *P*-value 0.05, calculated from three individual experiments. Data were analyzed using SA Bioscience templates. Heat maps were drawn and higherarchical clusters were represented using Multi Experiment Viewer. Predicted gene interaction network was obtained from STRING database with the input of experiment-derived genes as query.
