# Supplementary material for: Arsenic-induced instrumental genes of apoptotic signal amplification in death-survival interplay
Source: Cell Death Discov. 2016 Oct 17;2:16078–. doi: 10.1038/cddiscovery.2016.78 (PMC5066266; doi:10.1038/cddiscovery.2016.78)
Supplement: Supplementary Data 1 [file cddiscovery201678-s1.ppt]

## Slide 1
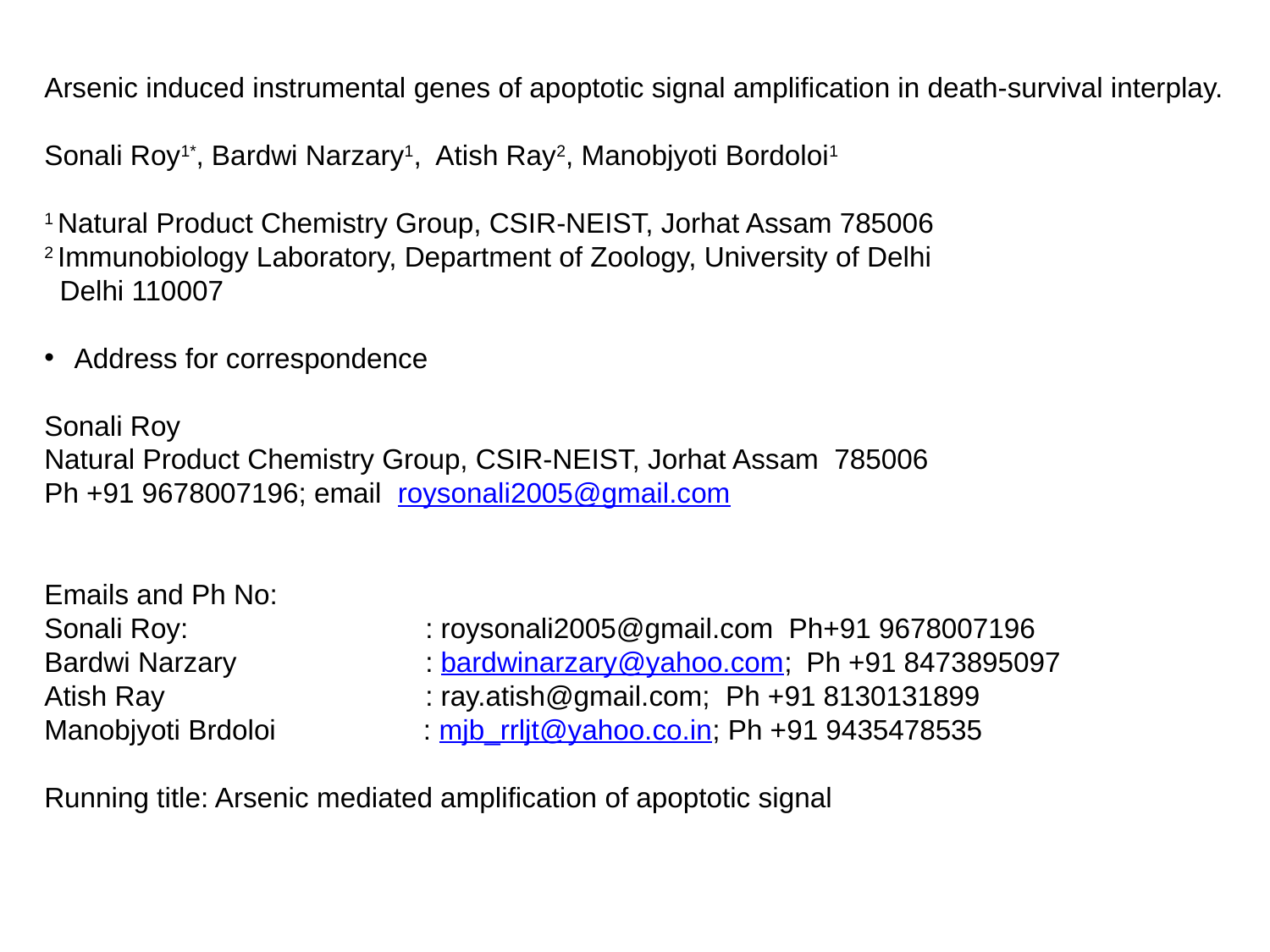

Arsenic induced instrumental genes of apoptotic signal amplification in death-survival interplay.
Sonali Roy1*, Bardwi Narzary1, Atish Ray2, Manobjyoti Bordoloi1
1 Natural Product Chemistry Group, CSIR-NEIST, Jorhat Assam 785006
2 Immunobiology Laboratory, Department of Zoology, University of Delhi
 Delhi 110007
Address for correspondence
Sonali Roy
Natural Product Chemistry Group, CSIR-NEIST, Jorhat Assam 785006
Ph +91 9678007196; email roysonali2005@gmail.com
Emails and Ph No:
Sonali Roy: 		: roysonali2005@gmail.com Ph+91 9678007196
Bardwi Narzary		: bardwinarzary@yahoo.com;	Ph +91 8473895097
Atish Ray 		: ray.atish@gmail.com; Ph +91 8130131899
Manobjyoti Brdoloi	 : mjb_rrljt@yahoo.co.in; Ph +91 9435478535
Running title: Arsenic mediated amplification of apoptotic signal

## Slide 2
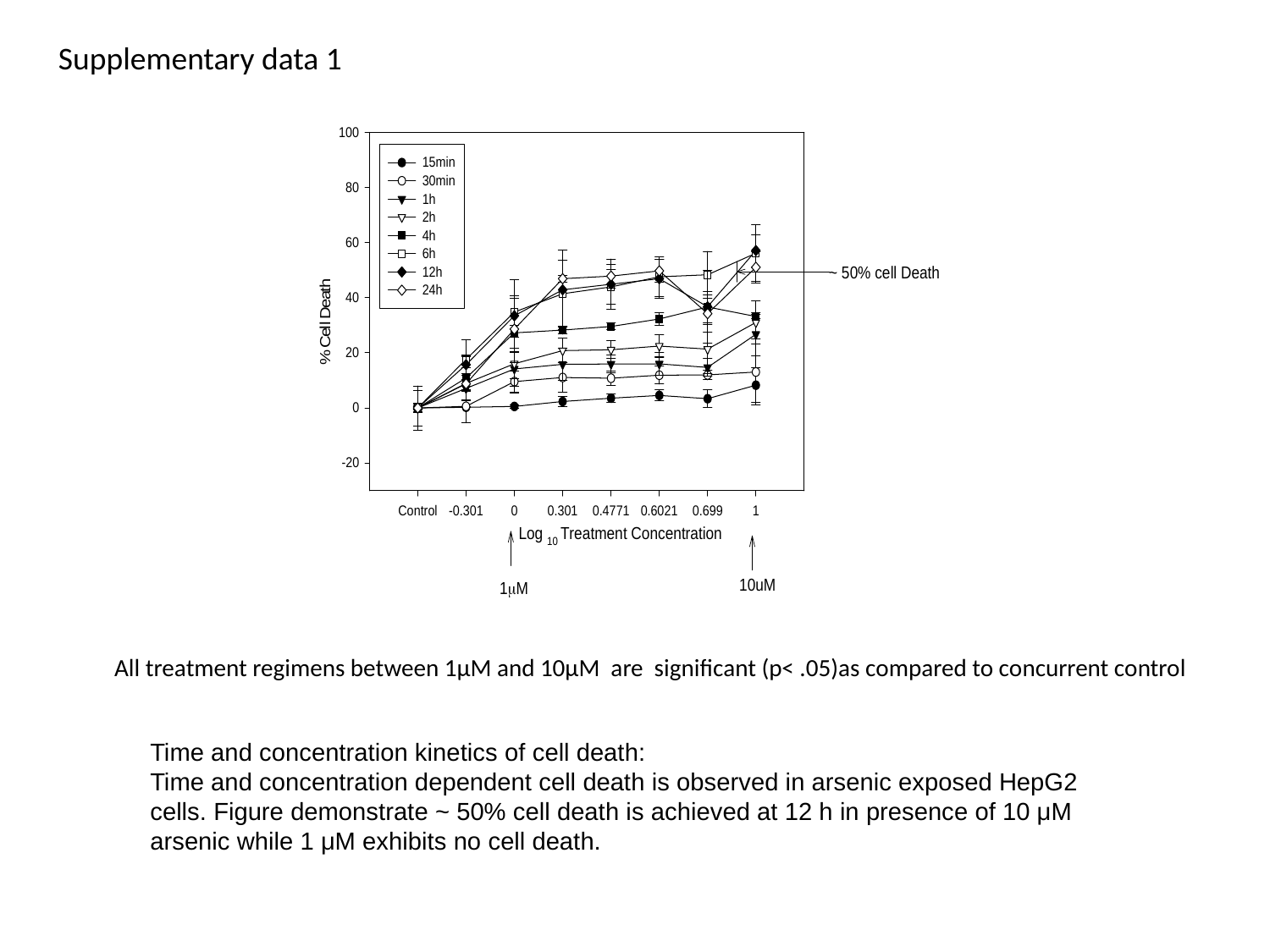

Supplementary data 1
All treatment regimens between 1µM and 10µM are significant (p< .05)as compared to concurrent control
Time and concentration kinetics of cell death:
Time and concentration dependent cell death is observed in arsenic exposed HepG2 cells. Figure demonstrate ~ 50% cell death is achieved at 12 h in presence of 10 μM arsenic while 1 μM exhibits no cell death.
